# Short-Term Impact of Staying Home on Bone Health in Patients With Osteoporosis During a State of Emergency Declaration Due to COVID-19 in Kanagawa, Japan

**DOI:** 10.7759/cureus.10278

**Published:** 2020-09-06

**Authors:** Yuji Yokozeki, Kentaro Uchida, Masayuki Miyagi, Kosuke Murata, Tomohisa Koyama, Akiyoshi Kuroda, Ayumu Kawakubo, Yuta Nanri, Gen Inoue, Masashi Takaso

**Affiliations:** 1 Department of Orthopaedic Surgery, Kitasato University School of Medicine, Sagamihara, JPN; 2 Department of Orthopaedic Surgery, Kitasato University, School of Medicine, Sagamihara, JPN; 3 Medical Sciences Research Institute, Shonan University, Chigasaki, JPN; 4 Department of Rehabilitation, Kitasato University Hospital, Kitasato University Hospital, Sagamihara, JPN

**Keywords:** covid19, stay-at-home, bone, osteoporosis

## Abstract

Background

On April 16, 2020, the Japanese government declared a state of emergency due to the spread of COVID-19 infection, leading prefectural governors to announce a stay-at-home order for 39 days until May 25, 2020. As physical inactivity is a risk factor for osteoporosis, we investigated the short-term impact of the stay-at-home order on bone health among patients with osteoporosis in our hospital in Kanagawa prefecture.

Methods

Thirty patients with osteoporosis with no delays in their regular medication who received care at our hospital’s osteoporosis outpatient clinic within one month after the end of the state of emergency were included. Lumbar spine and femur proximal bone mineral density (BMD) were measured at the last follow-up date (May 25 to June 30, 2020; 0M) and six (6M) and 12 months (12M) before the last follow-up using dual-energy X-ray absorptiometry. Bone alkaline phosphatase (BAP), Tartrate-resistant Acid Phosphatase 5b (TRACP5b), calcium and phosphorus were assessed at the same time points.

Results

Serum BAP concentrations were significantly lower at 0M than 12M (p=0.040), but were comparable between 0M and 6M (p=0.527). Serum TRACP5b was significantly lower at 6M than 12M (p=0.009), but was similar between 0M and 6M (p=1.000). Serum calcium and phosphorus did not differ among the time points (p=0.516 and p=0.358, respectively). Similarly, lumbar spine and femoral neck BMD were comparable (p=0.679 and p=0.076, respectively).

Conclusion

Bone health in patients with osteoporosis was maintained during the short-term COVID-19 stay-at-home order among patients who experienced no delays in medication. However, larger and long-term studies are needed.

## Introduction

In December 2019, a series of pneumonia cases of unknown cause emerged in Wuhan, Hubei, China, with clinical presentation resembling viral pneumonia [1]. Deep sequencing analysis of lower respiratory tract samples traced the cause to a novel coronavirus, named 2019 novel coronavirus (2019-nCoV). The virus, which was later classified and renamed severe acute respiratory syndrome coronavirus 2 (SARS-CoV-2), has since spread around the world, forcing governments to impose lockdown orders, to limit movement and activity among communities while allowing essential organizations/services to function, with the aim of preventing or reducing the spread of COVID-19, the disease caused by SARS-CoV-2. In Japan, the national government issued a state of emergency declaration on April 16, 2020. As such, prefectural governors requested all residents to stay at home unless they were performing essential tasks, although there would no legal penalties for those who disobeyed the order. While the national government made no mention of the duration of the state of emergency, prefectural governors of Tokyo, Kanagawa, Saitama, and Chiba prefectures announced that the stay-at-home orders would remain in force for 39 days until May 25, 2020.

Osteoporosis is a major health problem worldwide. The disease is characterized by low bone mass and micro-architectural deterioration of bone tissue, leading to enhanced bone fragility and a consequent increase in fracture risk [2]. Physical activity is a key factor necessary for maintaining bone mass, with physical inactivity being a potential cause of bone loss [3-5]. Thus, reduced physical activity due to the COVID-19 stay-at-home order may affect bone health in patients with osteoporosis.

We investigated the short-term impact of staying home on bone health among patients with osteoporosis during the COVID-19 state of emergency in our hospital in Kanagawa prefecture.

## Materials and methods

Ethics

This study received ethical approval from the Ethics Review Board of Kitasato University (approval number: B20-132). Informed consent was obtained in the form of opt-out on the website.

Patients

Thirty patients with osteoporosis (five males and 25 females) aged 71.9 ± 14.0 years (mean ± standard deviation) who received care at Kitasato University Hospital’s osteoporosis outpatient clinic within one month after the end of the state of emergency declaration were included. Of the 30 patients, 13 patients (43.3%) had prevalent fractures. All patients had been receiving osteoporosis medication without delay for at least one year in our hospital (Table 1). Patients with changes to the contents of their medication during the one-year period were excluded.

**Table 1:** Osteoporosis medications used by patients in this study SERM, selective estrogen modulator

	Number of patients	Active vitamin D analogue (with/without)
Denosmab	12	5/7
Bisphosphonate		
Oral	5	3/2
Intravenous	5	2/3
Active vitamin D analogue	3	3/0
Teriparatide	2	0/2
SERM	3	2/1

Assessments

**Figure 1:**
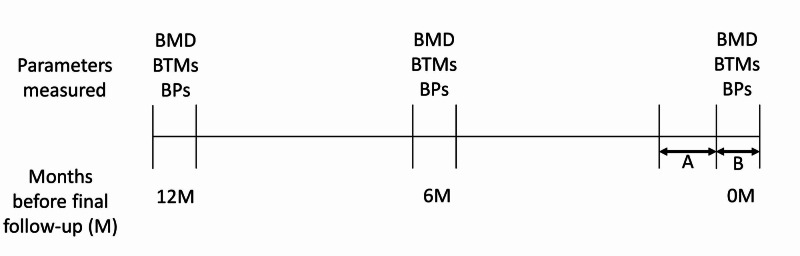
Scheme of study protocol A: Duration of stay-at-home order (April 16 to May 25, 2020), B: duration of final follow-up (May 26 to Jun 30, 2020). BTMs, bone turnover markers; BPs, biochemical parameters; BMD, bone mineral density.

Patients’ bone mineral density (BMD) was measured in the lumbar spine (L2-L4) and femoral neck using dual-energy X-ray absorptiometry (Hologic Inc., Bedford, MA) at the last follow-up date (May 25, 2020 to June 30, 2020; 0M) and six (6M) and 12 months (12M) before the last follow-up (Figure 1). Bone alkaline phosphatase (BAP), Tartrate-resistant Acid Phosphatase 5b (TRACP5b), calcium (Ca) and phosphorus (P) were assessed at same time points.

Statistical analysis

 All statistical analyses were performed using Statistical Package for the Social Sciences (SPSS), version 25.0. Analysis of variance with repeated measures and a Bonferroni post hoc test were used to compare the three groups. Statistical significance was defined as p<0.05.

## Results

Changes in BMD and serum bone metabolism markers

**Figure 2:**
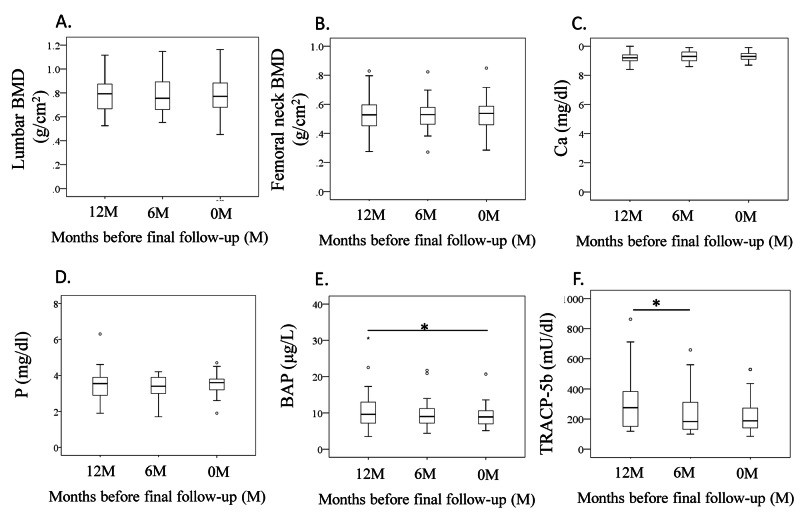
Change in bone mineral density and serum bone metabolism markers (A) Femoral neck BMD, (B) lumbar BMD, (C) Calcium (Ca), (D) phosphorus (P), (E) bone alkaline phosphatase (BAP), (F) TRACP-5b. *p<0.05. BMD, bone mineral density.

BMD in the lumbar spine and femoral neck did not differ among the three time points (p=0.679 and p=0.076, respectively; Figure 2A, B). Serum Ca and P levels did not differ among the three time points (p=0.516 and p=0.358, respectively; Figure 2C, D).Serum BAP concentrations were significantly lower at 0M than 12M (p=0.040; Figure 2E), but were comparable between 0M and 6M (p=0.527). Serum TRACP5b concentrations were significantly lower at 6M than 12M (p=0.009; Figure 2F), but were similar between 0M and 6M (p=1.000).

## Discussion

Several scientific articles have raised an alarm on the potential detrimental effects of the COVID-19 public health emergency on patients with osteoporosis, as a reduction in bone density scanning and decreased treatment can elevate fracture risk. To examine the association of physical activity with fracture risk [6-9], we evaluated the impact of reduced activity due to the stay-at-home order during the COVID-19 state of emergency on patients with osteoporosis who experienced no delays in their regular medication. Mobile-based mobility analysis showed that human mobility decreased by around 50% in Tokyo during the state of emergency [10]. However, no changes were observed in BMD or bone metabolism markers for a six-month duration that included the 39-day stay-at-home period. Previous studies have observed a reduction in BMD among patients confined to long-term bed rest [11,12], with a decrease in BMD initially observed 60 days after bed rest [12]. In contrast, changes in bone metabolic markers are reportedly detectable early after initiation of bed rest [3,11]: serum Ca levels increase 19 days after bed rest and serum BAP and TRAP-5b increase 12 days after bed rest in healthy men [11]. In contrast, a recent study reported an increase in bone resorption markers, Ca and P, in younger men (23±3 years), but not in older men (60±2 years) during 14 days of bed rest, suggesting that older men are not at an elevated, but may actually be at a reduced, risk of bone loss when immobilized [3]. Together, these previous findings and those of the present study suggest that staying home for a short period of time has little effect on bone metabolism in elderly patients with osteoporosis.

Previous studies have reported that bisphosphonates prevent bone loss during bed rest and space flights [13,14]. Combination treatment comprising an anti-bone resorptive drug and active vitamin D analogue has been shown to prevent bone loss in the hind limbs of immobilized rats [15]. In our study, 93% of patients were receiving an anti-resorptive drug including denosmab, a bisphosphonate, and selective estrogen modulator. In addition, most patients were receiving an active vitamin D analogue and another osteoporosis drug, unless they had hypercalcemia or other complications. It remains unclear which of these osteoporosis drugs are effective for preventing bone loss. Our results indicate that bone health was maintained during the short-term stay-at-home order among patients who experienced no delays in their regular medication.

Patients with osteoporosis are likely to be at high risk of sequelae after contracting COVID-19. Several studies have recommended a temporary transfer to oral bisphosphonate for patients with osteoporosis because of the need for face-to-face clinical intervention for intravenous administration or subcutaneous injection of osteoporosis drugs [16,17]. We continued to administer osteoporosis drugs such as denosmab and teriparatide because the number of COVID-19 patients decreased temporarily after the end of the state of emergency in Kanagawa prefecture. However, temporary transfer to oral bisphosphonate may be necessary when COVID-19 cases rise again in Japan.

While we observed that staying home for a short period of time had little impact on bone health, prolonged or repeated stay-at-home orders due to COVID-19 spread may affect bone health, even among patients who continue to receive their regular medications. Patients with osteoporosis are advised to engage in regular weight-bearing exercise to improve their strength, balance, posture and reduce the risk of falls [18,19]. Home-based exercise programs have been shown to improve the quality of life of older individuals, may improve muscle mass and are feasible [20]. Prescription of a suitable home-based program is necessary to maintain bone health among patients with osteoporosis in the future.

There were two main limitations in the present study. First, we did not examine the activity of participants during declaration. Second, there is no control group remaining active.

## Conclusions

In conclusion, we evaluated the short-term impact of a stay-at-home order on bone health among patients with osteoporosis during a state of emergency due to COVID-19 in Kanagawa, Japan. Our results indicate that bone health was maintained during the short-term stay-at-home order among patients who experienced no delays in their regular medication. However, further investigations with larger sample size and long-term observations are needed.
